# High precision robust control design of piezoelectric nanopositioning platform

**DOI:** 10.1038/s41598-022-14332-5

**Published:** 2022-06-20

**Authors:** Huan Feng, Aiping Pang, Hongbo Zhou

**Affiliations:** School of Electrical Engineering, Guizhou University, Guiyang, 550025 China

**Keywords:** Engineering, Electrical and electronic engineering, Mechanical engineering

## Abstract

The piezoelectric nanopositioning platform requires extremely accurate tracking during the task, while the model uncertainty caused by load variations requires strong robustness of the system. The high accuracy and robustness in the control design are coupled to each other, making it difficult to achieve both optimally at the same time. In addition, the system itself has a weakly damped resonant mode, which makes it extremely difficult to control the piezoelectric nanopositioning platform while suppressing the inherent resonance of the system as well as meeting the requirements for robustness and high accuracy. For the multi-performance integrated control problem of piezoelectric nanopositioning platform, this paper gives two kinds of control designs (integral resonance control (IRC) and H∞ control) satisfying accuracy requirements and robustness, and carries out simulation study and comparative analysis with positive position feedback control (PPF). Simulation results show that the H∞ control strategy given in this paper has the smallest tracking error compared to PPF and IRC under 5, 10 and 20 Hz input grating scan signals, though it has a higher order, with better robustness to mechanical load variations and high frequency signal perturbations in the 0–1000 g load range.

## Introduction

With the introduction of the first scanning tunneling microscope (STM), atomic force microscope (AFM) and scanning probe microscope (SPM), the development of nanotechnology has entered a new era, and mankind has started to explore and innovate continuously in the microscopic world, all thanks to the development of piezoelectric nanopositioning systems. Nowadays, this positioning system has been widely used in high-precision fields such as micro-robotics, micro-assembly, micro-assembly, micro-lithography, micro-machining and micro-scanning^1–5^. Piezoelectric ceramics are commonly used to drive these nanopositioners due to their advantages of fast kinetics, high output force, and high sub-nanometer resolution^6^. In previous control studies for piezoelectric-driven nanopositioning systems, the operating bandwidth of piezoelectric-driven nanopositioning systems was usually limited to 10–100 times lower than the lowest intrinsic resonant frequency of the system because the system has a weakly damped resonant mode. However, with the rapid development of nanotechnology, practical applications require higher and higher speed and accuracy for piezoelectric nanopositioning systems. As in the life sciences, some biological samples to be scanned have very light dynamic behaviors, such as protein molecules, living cells, and so on, which typically change within milliseconds^7^, so it is not possible to suppress the resonant vibration of the system by limiting the input signal. In addition, in practical system modelling and control, there are various uncertainties such as external disturbances, environmental changes, time delays and other factors that can seriously affect the positioning accuracy of the system if not properly dealt with. Different control methods based on cybernetics and modeling theory are proposed for the resonant vibration, high bandwidth tracking and robustness problems of piezoelectric nanopositioning systems. Control methods based on feedback architectures are widely used because of their robustness to external disturbances and model uncertainties^8^, such as adaptive control^9^ and linear quadratic Gaussian control^10^ proposed to reduce tracking errors in high-speed scanning tasks. However, these methods can only find controllers with good robustness when the *Q*-factor (represents the resonant frequency of the system relative to the bandwidth) of the system is low. As the damping ratio of the system becomes smaller if the *Q*-factor of the system becomes larger, it is difficult for the above methods to achieve high damping performance of the controller, which cannot guarantee the robustness and accuracy of the system^11^. To give priority attention to and solve the damping problem of piezoelectric-driven nanopositioning systems, model-based control strategies are proposed, such as using recursive delayed position feedback^12^ to attenuate the resonant modes of nanopositioning stages in the internal feedback loop, resulting in a neutral-type time-lag system; using robust mass dampers^13^ to significantly improve the platform resonant mode damping in industrial high-precision motion platform design; and using model-referenced control^14^ in the form of pole configurations, combined with integrator and filtering effects to reduce sensitivity to disturbances and uncertainties to achieve good tracking performance, etc.

In addition to the above work, negative imaginary number theory^15,16^ provides a solution that increases vibration mode damping while maintaining robustness to modal uncertainty and non-modal dynamics at the same time, and this solution is suitable for dealing with resonance problems of flexible structures with weakly damped modes. Damping controllers designed based on negative imaginary number theory improve the bandwidth of the piezoelectric nanopositioning platform with good performance while suppressing resonant modes, such as positive position feeding back (PPF)^17^, positive velocity position feeding back^18^, resonant control (RC)^19^, force integral feedback control, integral RC (IRC)^20,21^ and so on. All of the above controllers have low order, low computational complexity fixed structures, making them simple to design and implement. However, each of these controllers has its own drawbacks in applications. The PPF controller cannot achieve arbitrary configuration of second-order poles in the s-plane; the integral resonant control is designed to consider model uncertainty in the control design, and the system robustness needs to be improved; the force-integral feedback control has restricted application conditions and requires the introduction of force sensors, etc.

This paper addresses the inherent weakly damped resonant modes, model uncertainty and high-speed tracking problems of the piezoelectric-driven nanopositioning platform and, firstly, designs the IRC control structure by combining internal damping control, robust control and tracking control. The internal damping controller is designed to suppress the resonant modes according to the damping characteristics of the system; a robust controller is added to the internal damping controller to limit the bandwidth of the control system and improve the robustness of the system; and a tracking controller is used to improve the tracking accuracy and reduce the steady-state error. However, in high-speed scanning tasks under IRC control, the modelling uncertainty caused by load variations can reduce the robustness of the system and lead to poor performance. In order to enhance the robustness of the system in the high-speed scanning task with load variation, this paper uses three corresponding weighting functions to limit the control performance, and designs an H∞ controller that satisfies the requirements of bandwidth limitation, high-precision tracking, and strong robustness. The simulation shows that both controllers designed in this paper can meet the control requirements of the system when coping with the 5 Hz and 10 Hz scanning task, and have certain robustness for different mechanical load variations. The H∞ control has faster response speed and higher accuracy compared with the IRC control. Also in the relatively high frequency (20 Hz) scanning task, the H∞ controller is more robust for a wider mechanical load variations (0–1000 g) and high frequency signal perturbations while achieving higher accuracy and faster response times.

## Piezoelectric nanopositioning platform and its model

**Figure 1:**
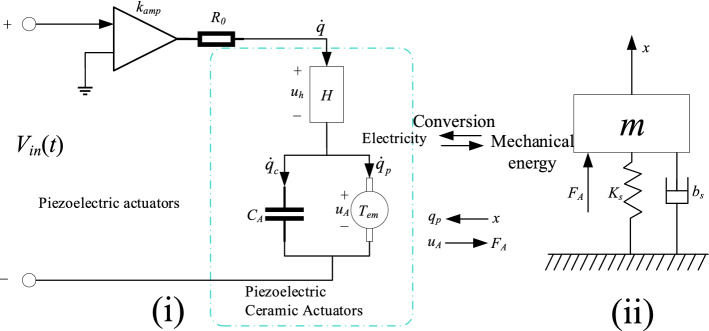
Schematic diagram of the piezoelectric positioning system.

Modelling the dynamics of piezoelectric nanopositioning systems is an essential prerequisite for gaining insight into system performance and exploring control algorithms for high-speed motion. The actual positioning of motion by a piezoelectric positioning system is a process of converting energy from electrical energy to mechanical energy. Starting with the excitation signal from the dSPACE controller, the electrical signal is amplified by voltage and drives a piezoelectric ceramic actuator. Due to the inverse piezoelectric effect of the piezoelectric ceramic, the actuator generates thrust, which drives the flexible mechanism to produce position movement. This energy conversion relationship relies on the coupling link between the piezoelectric actuator, the piezoelectric ceramic actuator and the platform mechanics in the piezoelectric positioning system. The piezoelectric positioning system is a relatively complex electromechanical coupled system, so the dynamic characteristics of the piezoelectric actuator, the piezoelectric ceramic actuator and the mechanical structure of the platform need to be considered comprehensively to establish a relatively accurate dynamics model. A schematic diagram of the piezoelectric positioning system^22^ is shown in Fig. 1, and a physical drawing of it can be found in the literature 23.

Figure 1(i) shows the equivalent circuit schematic of the piezoelectric actuator and piezoelectric ceramic actuator. Using Kirchhoff's law, the circuit models of the piezoelectric driver and piezoelectric ceramic actuator can be resolved as follows,

1 $$ R_{0} \dot{q}(t) + u_{h} (t) + u_{A} (t) = k_{amp} V_{in} (t) $$

2 $$ u_{h} (t) = H(q) $$

3 $$ q(t) = q_{c} (t) + q_{p} (t) $$

4 $$ u_{A} (t) = q_{c} (t)/C_{A} $$

5 $$ q_{p} (t) = T_{em} x(t) $$

where *V*_*in*_(*t*) is the input voltage , *k*_*amp*_ is the amplification factor of the piezoelectric actuator, *R*_0_ is the equivalent internal resistance of the drive amplifier circuit, *q* is the total charge applied to the piezoelectric ceramic actuator, and $\dot{q}$ is the current flowing through the circuit generated by the charge *q* . *u*_*h*_ represents the voltage of the piezoelectric hysteresis effect *H* , *C*_*A*_ represents the total capacitance of all piezoelectric ceramics, and *q*_*c*_ is the charge stored in capacitor *C*_*A*_. *T*_*em*_ represents the piezoelectric effect, and *u*_*A*_ is the voltage generated by the piezoelectric effect , and *q*_*p*_ is the charge caused by the piezoelectric effect. *x* is the output displacement produced by the active body under the thrust of the actuator.

Based on the kinematic relationship between the piezoelectric ceramic actuator, the flexible amplification mechanism and the movable body in the piezoelectric nanopositioning platform, the mechanical transmission of the piezoelectric nanopositioning platform can be simplified to a mass-spring-damping system, an equivalent mechanical dynamics model as shown in Fig. 1(ii). The equivalent mechanical model can be resolved according to Newton's laws of motion and piezoelectric effects as follows,

6 $$ F_{A} = T_{em} u_{A} (t) $$

7 $$ m\ddot{x}(t) + b_{s} \dot{x}(t) + k_{s} x(t) = F_{A} $$

where *m*, *k*_*s*_ and *b*_*s*_ denote the total mass, overall stiffness and damping coefficient of the active body of the piezoelectric nanopositioning platform, respectively, and *F*_*A*_ is the mechanical thrust generated by the piezoelectric ceramic actuator. It is worth mentioning that the model equations for the charge input and displacement output of the system can be derived from Eqs. (4), (6) and (7) as follows,

8 $$ m\ddot{x}(t) + b_{s} \dot{x}(t) + k_{s} x(t) = \frac{{T_{em} }}{{C_{A} }}q(t) $$

Equation (8) uses charge as the control input, and the hysteresis effect *H*(*q*) can be avoided by using charge control. However, in practical systems, voltage is usually used as the control input to drive the piezoelectric nanopositioning platform. if voltage control is used, Eqs. (1)–(7) can be combined to establish a comprehensive dynamics model of the system with respect to voltage input *u*_*in*_ and displacement output *x*.

9 $$ \dddot x(t) + a_{2} \ddot{x}(t) + a_{1} \dot{x}(t) + a_{0} (t) = b_{1} u_{in} (t) - b_{0} H(q) $$

The parameters in the formula are expressed as follows:

$$ b_{0} = \frac{{T_{em} }}{{mR_{0} C_{A} }} $$

$$ b_{1} = \frac{{T_{em} k_{amp} }}{{mR_{0} C_{A} }} $$

$$ a_{0} = \frac{{R_{0} k_{s} C_{A} + (R_{0} - 1)T_{em}^{2} }}{{mR_{0} C_{A} }} $$

$$ a_{1} = \frac{{k_{s} C_{A} + T_{em}^{2} + b_{s} }}{{mC_{A} }} $$

$$ a_{2} = \frac{{b_{s} C_{A} + m}}{{mC_{A} }} $$

To avoid the non-linear effects caused by voltage hysteresis, a low-amplitude input voltage (a sinusoidal scanning input voltage with a constant amplitude of 200 mV between 0.1 and 500 Hz applied to the y-axis) was used in the literature^23^ during the identification of the actual system, avoiding hysteresis-induced distortions^24^ and obtaining an approximation of the dominant dynamics of the piezoelectric nanopositioning platform with a weakly damped mode as a second-order system, as shown in Eq. (10),

10 $$ G(s) = \frac{y(s)}{{u(s)}} = \frac{{\sigma^{2} }}{{s^{2} + 2\xi_{n} w_{n} s + w_{n}^{2} }} $$

where *s* is the Laplace operator of the continuous system, *y*[*µm*] and *u*[*V*] are the output displacement and the input drive voltage, respectively, *σ*^2^ is the low-frequency gain of this system, and *ξ*_*n*_ and $w_{n}$ are the damping coefficient and the intrinsic frequency of the system. In this system, *ξ*_*n*_ ≪ 1, which means that the resonant mode at $w_{n}$ is weakly damped.

In the actual modelling and control process, there are various uncertainties, the most influential of which is the uncertainty caused by different mechanical loads. The system identification parameters obtained for mechanical loads of 0 g (nominal system), 600 g and 1000 g respectively are shown in Table 1.

**Table 1:** System model under different mechanical loads.

Mechanical loads (g)	Transfer functions
0	$G_{0} (s) = \frac{{1.198 \times 10^{6} }}{{s^{2} + 110s + 1.673 \times 10^{6} }}$
600	$G_{1} (s) = \frac{{0.8235 \times 10^{6} }}{{s^{2} + 282s + 1.15 \times 10^{6} }}$
1000	$G_{2} (s) = \frac{{0.5442 \times 10^{6} }}{{s^{2} + 419s + 0.76 \times 10^{6} }}$

**Figure 2:**
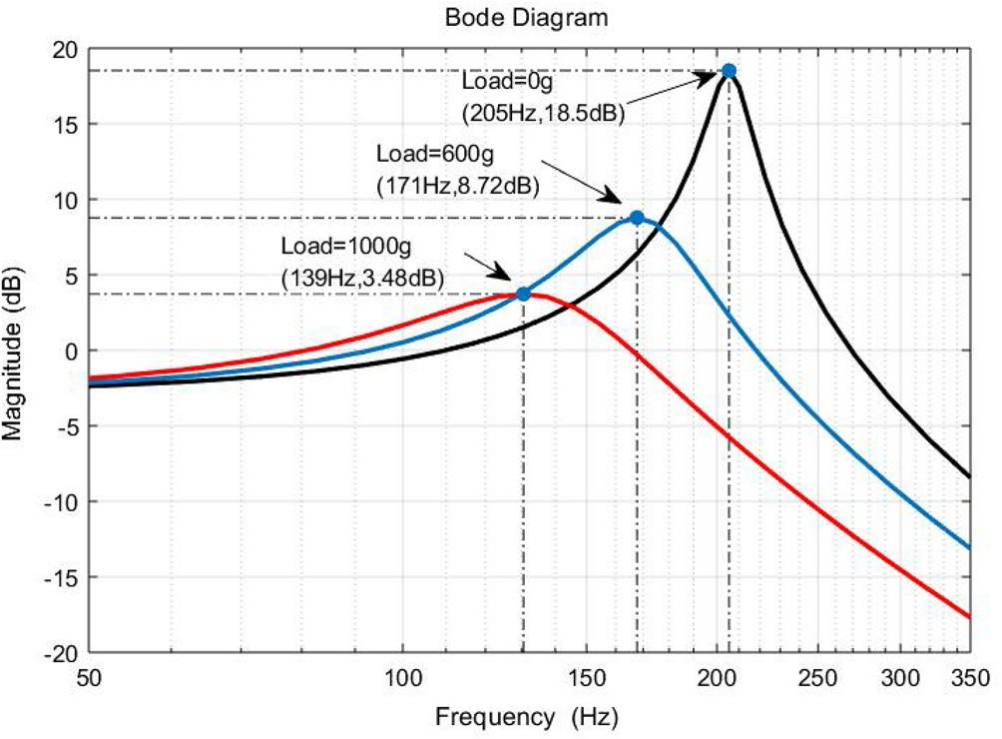
Open-loop Bode diagram of the system at mechanical loads of 0 g, 600 g and 1000 g.

The frequency response of the system under different mechanical loads is shown in Fig. 2. The inherent resonant mode of the nominal system occurs at 205 Hz with a resonance amplitude of 18.5 dB. While suppressing the inherent resonant mode of the system, the uncertainty of the model caused by the variation of the mechanical load cannot be ignored and will seriously affect the control accuracy of the system if not properly handled. Therefore, there is an urgent need to design a controller that meets the requirements of suppressing the inherent resonant modes and ensuring the robustness to achieve the task of scanning the platform with high accuracy.

## Control design

### Integral resonance control(IRC)

**Figure 3:**
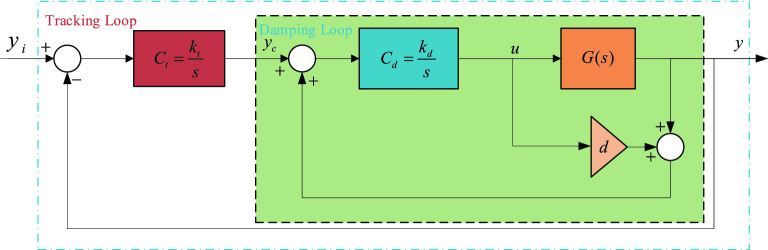
IRC control block diagram.

This section analyses a controller design approach based on the IRC method to meet the accuracy and robustness requirements of a piezoelectric driven nanopositioning platform, and the IRC control diagram is shown in Fig. 3. IRC contains two loops, an inner-loop positive feedback loop for damping control and an outer-loop negative feedback loop for improved tracking accuracy, where *G* is the system object, here the nominal model *G*_0_ without mechanical loads in Table 1 is used as the control object, *C*_*d*_ is the damping controller, *C*_*t*_ is the tracking controller, *d* is the feed-through term, *y*_*i*,_ *y*_*c*_ and *y* are the reference input, tracking controller output and system displacement output respectively, and *u* is the control input.

The inner loop from *y*_*c*_ to *y* is denoted as the damping loop, with a transfer function as following,

11 $$ T_{damp} (s) = \frac{{C_{d} (s) \cdot G(s)}}{{1 - C_{d} (s) \cdot (G(s) + d)}} $$

In the damping loop, the purpose of adding the feed forward term *d* is to generate a pair of zeros *z*_*1*_*, z*_*2*_ =$\pm jw_{z}$ in the root trajectory of the damping loop satisfying $\frac{{w_{n} }}{3} < w_{z} < w_{n}$. As the controller gain increases, the root trajectory starts at the natural pole and ends at the zero point induced by the addition of *d*. The feed-through term *d* can be taken as,

12 $$ d = - 2\frac{{\sigma^{2} }}{{w_{n}^{2} }} $$

The damping controller gain *k*_*d*_ is found to maximize the damping ratio of the damping loop, which can be calculated by the following equation,

13 $$ k_{{d|\xi_{\max } }} = \frac{1}{|d|}(w_{n} \cdot \sqrt {\frac{{w_{n} }}{{\sqrt {w_{n}^{2} + \sigma^{2} /d} }}} ) $$

where *ξ*_*max*_ is the maximum achievable damping ratio.

For tracking controller *C*_*t*_(*s*), the gain should meet following inequality:

14 $$ k_{t} \cdot k_{d} < - \frac{{\sigma^{2} + d \cdot w_{n}^{2} }}{{d^{2} }} $$

**Figure 4:**
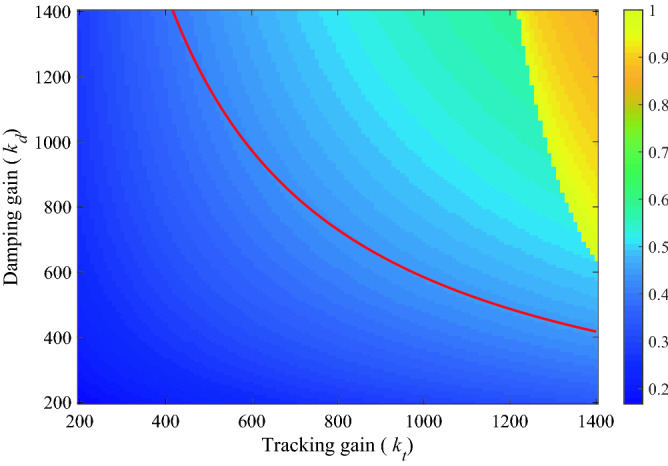
Normalized bandwidth diagram of the system under (k_d_/k_t_) parameter variation.

It is worth noting that only the internal damping loop in (12) is related to the resonant mode of damping (11). The outer-loop tracking controller is used to minimize the tracking error, especially in the low frequency region. For the selection of both gains as shown in Fig. 4, the combination of the two gains (*k*_*d*_ /*k*_*t*_) must lie in the region below the solid red line to ensure stability.

In summary, according to (13) and (14), the parameters of the IRC controller can be analytically derived as,

$$ d = - 1.43,\;\;C_{d}^{IRC} = \frac{980}{s},\;\;C_{t}^{IRC} = \frac{430}{s} $$

For the selection and adjustment process of the standard IRC parameters, *k*_*d*_ and *k*_*t*_ are fixed once they are selected. Therefore, in this design method and the improved analytical design method^20,21^, neither the trial-and-error method nor the analytical method takes into account the system uncertainty caused by mechanical load variations, so there is room for improvement in the adaptability of the standard IRC and robustness needs to be improved.

### Robust H∞ control design

#### System performance requirements

**Figure 5:**
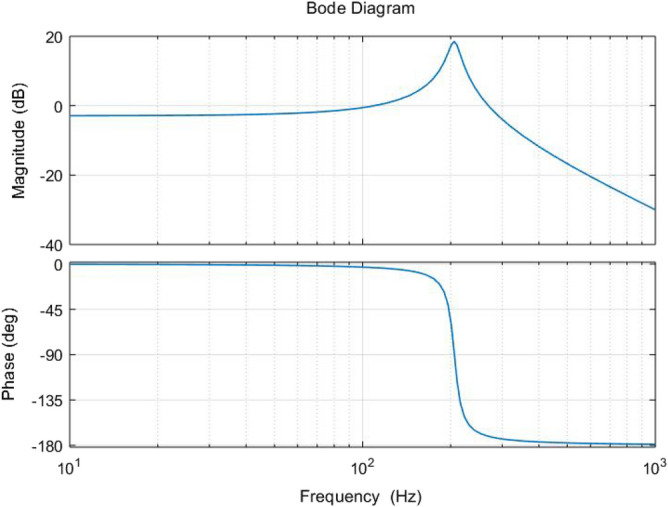
Open-loop Bode diagram for nominal system G_0_.

The open-loop Bode diagram of the nominal mathematical model *G*_0_ under a mechanical load of 0 g in Table 1 is shown in Fig. 5, from which it can be seen that the system has an inherently low-damped resonant mode at 205 Hz, with a resonant peak of 18.5 dB. When the excitation signal input to the system has a high-frequency component close to the resonant frequency of the platform, it will excite the resonant vibration of the system, and serious resonance will even damage the system hardware. Therefore, one of the performance requirements of the system is to suppress the inherent resonant modes of the system.

In addition, the system's surroundings changing or items with different loads scanned at high speed cause model uncertainty of the system, which can be considered as an un-modelled dynamics module, namely the uncertainty of the system. In the study of this control problem, only the ontology module of the piezoelectric nanopositioning platform is modelled, all others are considered as uncertainties of the system. The uncertainty of the system is expressed in terms of multiplicative uncertainty.

15 $$ G_{{{\text{actual}}}} (s) = G(s,\delta )[1 + \Delta G(s)] $$

where $\delta$ characterizes the structural uncertainty of the system, $\Delta G$ epresents the non-structural uncertainty of the system, *G* is the mathematical model constructed from the problem under study, and *G*_actual_ is the real physical model. Therefore, the second performance requirement of the system is robust stability.

**Figure 6:**
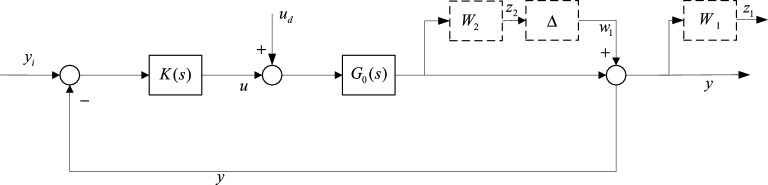
Block diagram of the control structure of H∞.

For the above performance requirements, the H∞ theory is used for control design. The control structure is shown in Fig. 6, where: *G*_0_(*s*) is the controlled object, *K*(*s*) is the controller, $\Delta$ ertcharacterizes the unstructured uncainty and structural uncertainty of the system; *W*_1_ and *W*_2_ represent the system performance weighted, uncertainty weighted; *w*_1_ represents the modelling uncertainty, *z*_1_ is the system performance output and *z*_2_ is the system robust performance output; *y*_*i*_ is the reference input, *y* is the system output; *u* is the control input and *u*_*d*_ is the control input perturbation.

#### Weighting function selection

According to the above analysis, the performance requirements of control system are the suppression of resonant modes and robust stability requirements of the system. In order to achieve these performance requirements, the design of the weighting function is particularly critical, and the selection of appropriate parameters to optimize the performance has become a hot topic of research for many scholars^25^. In the following, appropriate weighting functions are selected for each of the above two performance requirements in order to solve the controller.

The weighting function $W_{{1}} {(}s{)}$ mainly shows the disturbance decay performance, so it is required that controller should contain an integration link in order to ensure high precision tracking, as well as the system is expected to decay faster outside the bandwidth. So the weight function $W_{{1}} {(}s{)}$ can be designed as,

16 $$ W_{1} \left( s \right) = \frac{\rho }{{s\left( {s + 1000} \right)}} $$

where *ρ* is the parameter to be optimized.

Meanwhile, according to the open-loop amplitude-frequency characteristics of the piezoelectric-driven nanopositioning system as shown in Fig. 5, for the inherent resonant mode of the system at 205 Hz, sufficient attenuation suppression should be provided for this inherent resonant mode. Thus, the weighting function $W_{{1}} {(}s{)}$ must have good trap performance, which requires the addition of trap filtering characteristics in $W_{{1}} {(}s{)}$. To sum up, set the performance weighting function $W_{{1}} {(}s{)}$ as follows,

17 $$ W_{1} \left( s \right) = \frac{{\rho \left( {s^{2} + 2\xi_{2} \omega_{n} s + \omega_{n}^{2} } \right)}}{{s\left( {s + 1000} \right)\left( {s^{2} + 2\xi_{1} \omega_{n} s + \omega_{n}^{2} } \right)}} $$

For the control design of such weakly damped systems, it is advisable to design the system parameters in accordance with the lowest values, which can maintain the performance of the designed controller to a certain extent when the parameters become larger. The minimum damping ratio of the system is 0.0427 and the minimum resonant frequency is 139 Hz, with a corresponding amplitude of 3.48 dB. The center frequency of the trap filter is determined to be 139 Hz based on the damping ratio and trap frequency. Due to the different frequencies of the inherent resonant modes of the system under different loads, the filter width of the trap filter needs to have a certain margin. The performance weighting function $W_{{1}} {(}s{)}$ for the inherent resonant mode attenuation with trap filtering characteristics is shown in the following equation.

18 $$ W_{{1}} {(}s{) = }\frac{{\rho (s^{2} + 2 \times 0.04 \times 873s + 873^{2} )}}{{s\left( {s + 1000} \right)(s^{2} + 2 \times 1 \times 873s + 873^{2} )}} $$

**Figure 7:**
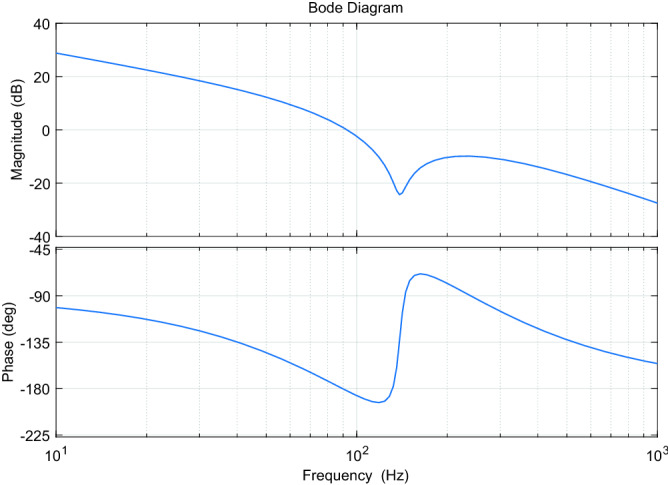
Bode diagram of the weighting function $W_{{1}} {(}s{)}$.

Its Bode plot (*ρ* = 1750) is shown in Fig. 7, and its amplitude-frequency characteristics satisfy the performance requirements.

Since the performance weight function $W_{{1}} {(}s{)}$ contains a pure integral link, to avoid the influence of the uncontrollable zero-pole on solving the H∞ controller, a small regressive momentum needs to be introduced at the pole to make it move off the imaginary axis into the left half-plane. The final $W_{{1}} {(}s{)}$ design is shown below,

19 $$ W_{{1}} {(}s{) = }\frac{{1750(s^{2} + 2 \times 0.04 \times 873s + 873^{2} )}}{{(s + 0.01)(0.001s + 1)(s^{2} + 2 \times 1 \times 873s + 873^{2} )}} $$

Considering the possibility of uncertainties in the system caused by various un-modelled dynamics, the weighting function $W_{2} (s)$ is chosen as a limit to the system bandwidth to ensure the robustness. The bandwidth of the system is limited to less than the minimum resonant mode frequency, and the closed-loop characteristics after the bandwidth are required to decay by − 40 dB/ dec. $W_{2} (s)$ is taken as $W_{2} (s) = \left( \frac{s}{800} \right)^{2}$ in this paper. In addition, because of the requirement of rank in the H_∞_ solution process, it is necessary to ensure that the weighting function is rational and true, so a small time constant is added to keep fractions of the same order. The final robust stability weighting function $W_{2} (s)$ can be chosen as,

20 $$ W_{2} (s) = \frac{{s^{2} }}{{800^{2} \left( {0.00001s + 1} \right)^{2} }} $$

Meanwhile, due to the requirement of the generalized controlled object for rank in H_∞_ control theory, the weighting function is an additional input channel introduced to satisfy the rank requirement, chosen as $W_{3} = 10^{ - 6}$.

#### H∞ controller

After iterative calculations, the final H_∞_ controller is obtained at the performance-optimized solution $\gamma = 1.3508$ as follows,

21 $$ K_{\infty } (s) = \frac{{ - 5.208 \times 10^{9} (s + 1220)(s + 1 \times 10^{5} )(s^{2} + 1754s + 9.226 \times 10^{5} )(s^{2} + 75.03s + 7.629 \times 10^{5} )}}{{(s + 6.024 \times 10^{7} )(s + 0.01)(s^{2} + 2374s + 1.374 \times 10^{6} )(s^{2} + 1746s + 7.621 \times 10^{5} )(s^{2} + 1.046 \times 10^{6} s + 5.473 \times 10^{11} )}} $$

Omitting the higher order terms with poles of 10^5^ or more, and then removing the additional regression term (0.01) from the poles where the rank requirement is considered in the design process, the simplified H_∞_ controller is simplified as ,

22 $$ K_{\infty } (s) = \frac{{ - 9.9229 \times 10^{11} (s^{2} + 1013s + 4.338 \times 10^{5} )(s^{2} + 110.2s + 1.673 \times 10^{6} )}}{{s(s + 1869)(s^{2} + 848.4s + 3.6 \times 10^{5} )(s^{2} + 1.548 \times 10^{6} s + 1.198 \times 10^{12} )}} $$

### Experimental simulation analysis

Addressing the resonance and robustness of piezo-driven nanopositioning platforms, the PPF control and the IRC control, H∞ control designed in ‘‘Control design’’ Section were simulated and analyzed for comparison respectively. As piezoelectric nanopositioning stages typically operate over a range of tens or even hundreds of microns, the reference input signal was set to be a triangular wave grating scan signal (100 μm amplitude) with different frequencies for the comparative analysis. In addition, the robustness of the system was verified under a set of mechanical loads (including 0 g, 600 g and 1000 g) and high frequency signal interference.

#### Grating tracking experiment results

**Figure 8:**
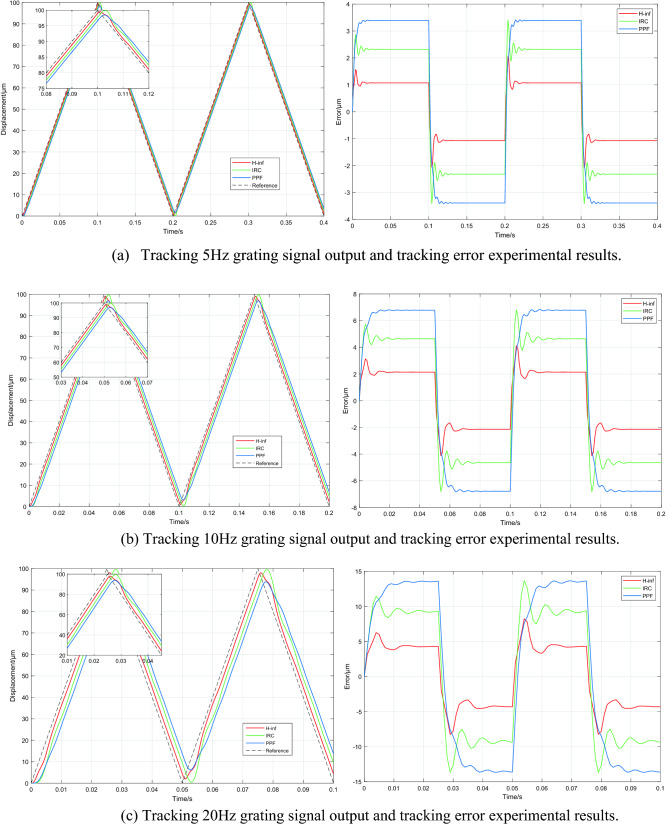
System tracking results for 5, 10 and 20 Hz grating input signals at 0 g mechanical load.

To evaluate the tracking performance of the three controllers, a set of grating scans at 5, 10 and 20 Hz were fed into the platform. The tracking outputs of the PPF, IRC and H∞ controllers for different frequencies of the grating input signals without high frequency disturbance signals are shown in Fig. 8, and the root mean square error (RMSE) of tracking are shown in Table 2. Under a mechanical load of 0 g, all three controllers tracked the reference input well. The values of RMSE are less than 6.781 μm when tracking the grating signal at 5 Hz and 10 Hz, particularly, the H∞ controller designed in this paper having the smallest RMSE value (less than 2.143 μm) among the three controllers. At 20 Hz, RMSEs of the PPF and IRC are 13.35 μm and 9.295 μm respectively, while the RMSE of the H∞ controller is 4.196 μm, achieving an improvement of 69% and 55% over the PPF and IRC respectively.

**Table 2:** System model under different mechanical loads.

Reference frequency(Hz)	Load(g)	RMSE with PPF(µm)	RMSE with IRC(µm)	RMSE with H∞(µm)
5	0	3.39	2.319	1.071
5	600	3.39	2.318	1.071
5	1000	3.391	2.314	1.071
10	0	6.781	4.637	2.143
10	600	6.781	4.644	2.142
10	1000	6.782	4.53	2.127
20	0	13.35	9.295	4.196
20	600	13.43	9.126	4.215
20	1000	13.34	8.996	4.579

#### Experimental results of robustness testing

**Figure 9:**
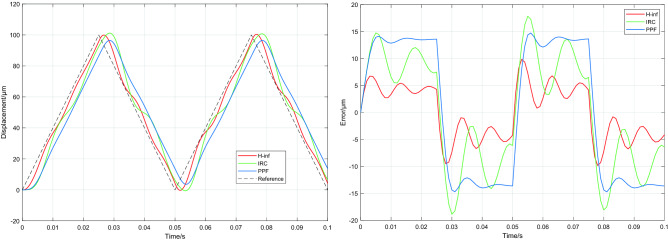
Results of the system tracking a 20 Hz grating scan signal at a mechanical load of 1000 g.

The maximum change in mechanical load in this experiment is 1000 g, when the first resonant frequency shifts from 205 to 139 Hz. Therefore, to verify the robustness of the PPF, IRC and H∞ controllers, the three controllers were simulated and analyzed for systems under different mechanical loads with the same frequency of grating scan signal input. The RMSEs of the three controllers with different mechanical loads are shown in Table 2. It can be seen that all three controllers have good robustness under different mechanical loads, but the H∞ controller has a higher tracking accuracy. The results of tracking the 20 Hz grating scan signal at a mechanical load of 1000 g are shown in Fig. 9, where the RMSEs of PPF and IRC are 13.34 μm and 8.996 μm respectively. In contrast, the RMSE of H∞ is only 4.579 μm, representing a 66% and 49% reduction over PPF and IRC respectively.

**Figure 10:**
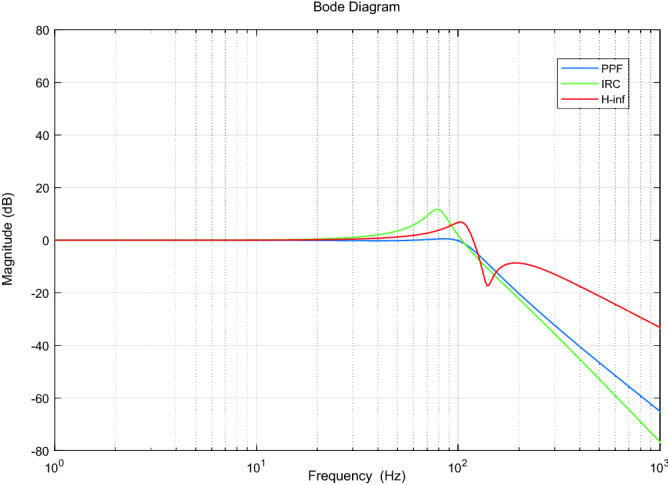
Closed loop Bode diagram for a system with a mechanical load of 1000 g with three controllers.

As the closed-loop Bode diagram of the system under a mechanical load of 1000 g shown in Fig. 10, the closed-loop bandwidth of the system is 115 Hz under the PPF controller, 112 Hz under the IRC and 123 Hz under the H∞ controller. Although the closed-loop bandwidths are similar for all three controllers, the H∞ controller has a slightly larger closed-loop bandwidth and the best response speed in comparison, as demonstrated in the grating signal tracking experiments, where the system tracked best under the H∞ controller.

#### High frequency signal disturbance performance testing

**Figure 11:**
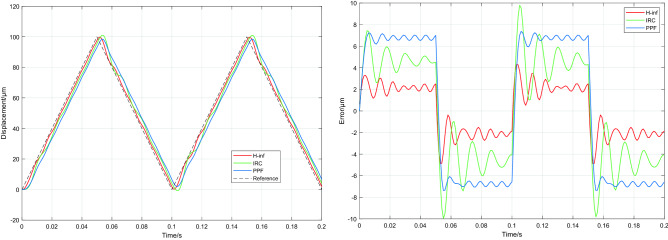
The system tracks a 10 Hz raster scan signal at a mechanical load of 1000 g and a perturbation frequency of 139 Hz.

A grating scan signal with a frequency of 10 Hz was fed into the closed-loop control system, and a high frequency disturbance signal close to the resonant frequency of the system (139 Hz) was added at a mechanical load of 1000 g. The RMSEs of the system under the PPF, IRC and H∞ controllers are 6.784 μm, 4.522 μm and 2.117 μm respectively, and the RMSE of H∞ was reduced by 69% and 53% compared to PPF and IRC respectively. In comparison of tracking results with high frequency sinusoidal disturbances (close to the resonant frequency of the system) shown in Fig. 11, it can be seen that there are large error fluctuations under IRC and large tracking errors under PPF. Conversely, H∞ control has good suppression and the best tracking effect, further verifying that the H∞ controller has good robustness.

## Conclusion

This paper addresses the comprehensive issues of inherent resonant modes, low bandwidth and uncertainties caused by mechanical load variations in high precision piezoelectric driven nanopositioning stages. The high accuracy requirements of the piezo-driven nanopositioning stage of less than 4.644 μm (tracking 5–10 Hz) with some robustness are achieved by designing an IRC control structure combining damping, robustness and tracking controllers, whose structure is simple but the robustness still needs to be improved under a wide range of mechanical load variation. Subsequently, the H∞ control is applied to the piezoelectric-driven nanopositioning platform, by analyzing the performance requirements to select the appropriate weighting function to give the H∞ controller with strong robustness, whose tracking accuracy (RMSE less than 2.143 μm at 5–10 Hz) and robustness performance are better than the IRC control structure. Finally, through the simulation of PPF, IRC and H∞ controllers for tracking different frequencies (5, 10, 20 Hz) of grating scanning signals and high frequency disturbance signals under different mechanical load variations, it is verified that the H∞ controller proposed offers a comprehensive superiority of accuracy, response speed and robustness.

## Data Availability

The data presented in this study are available on request from the corresponding author.
